# Supplementary material for: Learning-related contraction of gray matter in rodent sensorimotor cortex is associated with adaptive myelination
Source: eLife. 2022 Nov 9;11:e77432. doi: 10.7554/eLife.77432 (PMC9678357; doi:10.7554/eLife.77432)
Supplement: Figure 2—figure supplement 1—source data 1. — Whole-brain within-group analysis presenting the significant number of voxels (pFDR corr.<0.01 and < 0.001) together with the change in volume (mm3). [file elife-77432-fig2-figsupp1-data1.docx]

Figure 2- Supplementary Figure 1- source data 1. Effect of training on GMV and WMV in trained mice and the effect of time in non-trained control mice. Whole-brain within-group analysis presenting the significant number of voxels (*P*_FDR corr._ < 0.01 and < 0.001) together with the change in volume (mm^3^).

| Changes in GMV | | | | |
| --- | --- | --- | --- | --- |
|  | Trained mice (*n* = 39) | | Non-trained controls (*n* = 16) | |
|  | increase | decrease | increase | decrease |
| ***P*_FDR corr_ < 0.01** | | | | |
| Linear | - | - | 29327 (15.02) | - |
| Asymptotic | 2832 (1.45) | 139233 (71.29) | - | - |
| Quadratic | - | 39493 (20.22) | - | - |
| ***P*_FDR corr_ < 0.001** | | | | |
| Linear | - | - | 14316 (7.33) | - |
| Asymptotic | 1643 (0.84) | 97106 (46.72) | - | - |
| Quadratic | - | 15477 (7.91) | - | - |
|  | | | | |
| Changes in WMV | | | | |
|  | Trained mice (*n* = 39) | | Non-trained controls (*n* = 16) | |
|  | increase | decrease | increase | decrease |
| ***P*_FDR corr_ < 0.01** | | | | |
| Linear | - | - | - | - |
| Asymptotic | 26453 (13.54) | 2510 (1.29) | - | - |
| Quadratic | 91 (0.05) | - | - | - |
| ***P*_FDR corr_ < 0.001** | | | | |
| Linear | - | - | - | - |
| Asymptotic | 16590 (6.96) | 1196 (0.61) | - | - |
| Quadratic | - | - | - | - |
